# Altered Excitability and Glutamatergic Synaptic Transmission in the Medium Spiny Neurons of the Nucleus Accumbens in Mice Deficient in the Heparan Sulfate Endosulfatase *Sulf1*

**DOI:** 10.1523/ENEURO.0088-25.2025

**Published:** 2026-01-02

**Authors:** Ken Miya, Etsuko Suzuki, Kazuko Keino-Masu, Takuya Okada, Kenta Kobayashi, Toshihiko Momiyama, Masayuki Masu

**Affiliations:** ^1^Graduate School of Comprehensive Human Sciences, University of Tsukuba, Tsukuba 305-8575, Japan; ^2^Division of Biomedical Science, Department of Molecular Neurobiology, Institute of Medicine, University of Tsukuba, Tsukuba 305-8575, Japan; ^3^Department of Pharmacology, Jikei University School of Medicine, Minato-ku 105-8461, Japan; ^4^Section of Viral Vector Development, Center for Genetic Analysis of Behavior, National Institute for Physiological Sciences, National Institutes of Natural Sciences, Okazaki 444-8585, Japan

**Keywords:** knock-out mouse, medium spiny neuron, nucleus accumbens, *Sulf1*, whole-cell patch–clamp recording

## Abstract

Sulf1 is an extracellular sulfatase that regulates cell signaling by removing 6-*O*-sulfates from heparan sulfate. Although the roles of *Sulf1* in neural development have been studied extensively, its functions in the adult brain remain largely unknown. Here, we report the effects of *Sulf1* disruption on the neuronal properties of the medium spiny neurons (MSNs) in the nucleus accumbens (NAc) shell, one of the regions highly expressing *Sulf1*. We separately labeled MSNs expressing dopamine D1 receptors (D1-MSNs) or D2 receptors (D2-MSNs) by injecting adult male *Drd1-Cre* and *Drd2-Cre* mice with a Cre-dependent AAV vector expressing a red fluorescent protein, mCherry, and examined their electrophysiological properties by means of whole-cell patch–clamp recording. In the D2-MSNs, *Sulf1* disruption led to drastic changes in neural firing responses to depolarizing current injections: in the *Sulf1* knock-out mice, the rheobase was smaller than in the wild-type mice, but the number of action potentials elicited by depolarization did not increase at larger current injections. In the D1-MSNs, *Sulf1* disruption resulted in more depolarized resting membrane potentials and increase in the AMPA/NMDA ratio. These results suggest that *Sulf1* is essential for regulation of neuronal excitability and glutamatergic transmission of NAc MSNs in adult mice and implicate the potential roles of *Sulf1* in NAc circuit activity, reward-aversion behaviors, and psychiatric disorders such as schizophrenia and drug addiction.

## Significance Statement

Heparan sulfate (HS) plays critical roles in neural differentiation, axon guidance, synaptogenesis, and neurotransmission. Sulf1 is an extracellular sulfatase that removes 6-*O*-sulfate from HS, thereby regulating various cellular functions. Although its roles during development have been studied extensively, its functions in the adult brain remain largely unknown. Here, we examined the electrophysiological properties of medium spiny neurons (MSNs) in the nucleus accumbens shell of adult mice by means of whole-cell patch–clamp recording. We found that *Sulf1* disruption led to changes in neuronal excitability and glutamatergic transmission in MSNs. This study demonstrates the roles of the *Sulf1* gene in neuronal activities at the cellular level, providing an important clue toward understanding the functions of *Sulf1* in the adult brain.

## Introduction

Heparan sulfate proteoglycans (HSPGs) are glycoproteins ubiquitously present on the cell surface and in the extracellular matrix. They consist of a core protein and heparan sulfate (HS) sugar chains covalently attached to it. HSPGs interact with a large number of signaling molecules through HS, thereby regulating a wide variety of biological functions (Perrimon and Bernfield, 2000; Bishop et al., 2007). A number of previous studies have revealed that HSPGs play critical roles in cell differentiation, migration, axon guidance, synapse development, and synaptic functions in the nervous system (Holt and Dickson, 2005; Condomitti and de Wit, 2018; Zhang et al., 2018; Kamimura and Maeda, 2021). Genetic or enzymatic ablation of HS impaired synaptic transmission and plasticity (Irie et al., 2012; Minge et al., 2017), suggesting that HS is involved in the regulation of neurotransmission, but the underlying mechanisms remain largely unknown.

HS is a linear polysaccharide that consists of repeating disaccharide units composed of uronic acid and glucosamine. It has many sulfate groups necessary for interaction with its binding proteins. Sulf1 and Sulf2 are extracellular sulfatases that remove 6-*O*-sulfates from mature HS chains. They modulate various cellular functions by changing the interaction between HS and signaling molecules through 6-*O*-desulfation of HS (Dhoot et al., 2001; Morimoto-Tomita et al., 2002; Lamanna et al., 2007; El Masri et al., 2017). Studies using knock-out (KO) mice have revealed critical roles of *Sulfs* in normal development: although *Sulf1* or *Sulf2* single KO mice appear largely normal, *Sulf1/Sulf2* double KO mice die neonatally (Ai et al., 2007; Holst et al., 2007; Okada et al., 2017). *Sulf1/Sulf2* double KO mice have defects in esophageal innervation and axon guidance of the corticospinal tract (Ai et al., 2007; Okada et al., 2017). In both *Sulf1* and *Sulf2* single KO mice, development of oligodendrocyte precursor cells in the embryonic spinal cord is impaired (Touahri et al., 2012; Jiang et al., 2017). As compared with these well-studied roles of *Sulf1/Sulf2* genes in development, the functions of *Sulf1/2* in the adult brain remain largely unknown, with only one report published on synaptic and behavioral abnormalities in *Sulf1* KO mice (Kalus et al., 2009).

The nucleus accumbens (NAc) is a part of the basal ganglia located in the ventral forebrain. It plays important roles in appetitive and aversive responses (Floresco, 2015; Castro and Bruchas, 2019; Cox and Witten, 2019). The NAc receives glutamatergic inputs from the prefrontal cortex, ventral hippocampus, and basolateral amygdala, and the glutamatergic transmission is modulated by neurotransmitters such as dopamine from the ventral tegmental area (Britt et al., 2012; Floresco, 2015; Castro and Bruchas, 2019; Cox and Witten, 2019; Christoffel et al., 2021). More than 90% of neurons in the NAc are GABAergic medium spiny neurons (MSNs), which are subdivided into two groups: those expressing dopamine D1 receptors (D1-MSNs) and those expressing D2 receptors (D2-MSNs). A small number of MSNs express both D1 and D2 receptors. Many lines of previous clinical as well as animal model studies have revealed that the NAc is implicated in psychiatric disorders including schizophrenia, depression, obsessive–compulsive disorders, bipolar disorders, and drug addiction (Francis and Lobo, 2017; McCutcheon et al., 2019). Therefore, investigating the molecular mechanisms that regulate NAc activity is important from both basic and clinical perspectives.

In our previous study, we examined the expression patterns of *Sulf1/Sulf2* mRNA in the adult mouse brain systematically (Miya et al., 2021). Although *Sulf1* and *Sulf2* expressions overlap largely in the embryonic brain (Okada et al., 2017), they are mostly segregated in the adult brain. We found that *Sulf1* was highly expressed in the NAc, tail of the striatum, paraventricular nucleus of the thalamus, and prefrontal cortex (Miya et al., 2021). In addition, we showed that *Sulf1* expression was detected in most *Drd1-* or *Drd2*-expressing neurons in the NAc and other brain regions mentioned above (Miya et al., 2021). We thus thought it might be possible to clarify the roles of *Sulf1* in the adult brain by studying the effects of *Sulf1* KO on NAc MSNs in which *Sulf2* is not detected (Miya et al., 2021).

In this study, we examined the electrophysiological properties of MSNs in the NAc shell of adult mice by means of whole-cell patch–clamp recording. We show that *Sulf1* disruption leads to changes in neuronal excitability and glutamatergic transmission in MSNs. These data suggest that *Sulf1* is essential for regulation of neuronal activity and synaptic transmission of NAc MSNs in the mature brain.

## Materials and Methods

### Animals

Two- to twenty-seven-week-old male mice were used in this study. In situ hybridization data were obtained from three mice, and electrophysiological experiment data, from three to six mice. The experimenters were not blinded to genotype or cell identity. No statistical methods were used to predict the sample size before the study. All the animal experiments were approved by and performed according to the guidelines of the Animal Care and Use Committee of the University of Tsukuba (22-206, 22-211, 23-160, 23-165, 24-249, and 24-254) and of the Jikei University School of Medicine (2021-048C2 and 2022-009C1).

*Sulf1* KO mice, *Sulf1*^tm1Mmas^ (MGI 5318489), were generated by insertion of a cassette of stop-IRES-lacZ-poly(A) into the *Sulf1* gene using homologous recombination in ES cells, and *Sulf1* mRNA was shown to be completely abolished in the KO mice (Nagamine et al., 2012). *Drd1*-*Cre*, B6.FVB(Cg)-Tg(Drd1-cre)EY262Gsat/Mmucd (MGI 4358480), and *Drd2*-*Cre*, B6.FVB(Cg)-Tg(Drd2-cre)ER44Gsat/Mmucd (MGI 5003554; Gong et al., 2003; Heintz, 2004), maintained on a C57BL/6J background, were purchased from Mutant Mouse Resource and Research Centers. All these mice were backcrossed to C57BL/6N for 10 successive generations. *Sulf1*^−/−^;*Drd1*-Cre and *Sulf1*^−/−^;*Drd2*-*Cre* mice were generated by mating of mice carrying the *Sulf1* KO allele with *Drd1*-*Cre* or *Drd2*-*Cre* mice. Genotypes were determined by the use of genomic PCR with tail biopsy.

### Multiplex fluorescence in situ hybridization

Wild-type (WT) mice (15–19 weeks old) were deeply anesthetized with isoflurane and transcardially perfused with 4% paraformaldehyde in phosphate-buffered saline (PBS). The extracted brains were postfixed with the same solution at 4°C overnight. The brains were cryoprotected in 30% sucrose/PBS, embedded in Tissue-Tek OCT compound (Sakura Finetek), and stored at −80°C. Coronal brain sections (14 μm thick) were cut with a cryostat (CM 1850; Leica Biosystems) and collected onto MAS-coated slide glasses (Matsunami Glass Industry). Multiplex fluorescence in situ hybridization was performed by the use of an RNAscope Multiplex Fluorescent v2 Assay kit (Advanced Cell Diagnostics) according to the manufacturer's instructions. Specific probes for *Sulf1* (Mm-Sulf1 #495411), *Drd1* (Mm-Drd1a #406491-C2), and *Drd2* (Mm-Drd2 #406501-C3) were used. Signals were developed by means of a TSA Vivid Fluorophore kit 650 for *Sulf1*, 570 for *Drd1*, and 520 for *Drd2* (Tocris Bioscience). The sections were mounted with CC/Mount (Diagnostic BioSystems).

Images were acquired by the use of a laser scanning confocal microscope (LSM 700; Carl Zeiss) with a 10× objective lens for low-magnification images. Two regions of interest were selected from each of the three mice and analyzed by the use of a 20× objective lens and *Z*-stacking (1 μm intervals). Cells positive for *Sulf1*, *Drd1*, or *Drd2* were marked manually, and their overlapping was analyzed as described previously (Miya et al., 2021).

### Viral vectors

Adeno-associated virus (AAV) vectors were generated by use of the AAV Helper Free Expression System (Cell Biolabs) as reported previously (Sano et al., 2020). Briefly, HEK293T cells were transfected with the packaging plasmids (pAAV-RC5 and pHelper) and pAAV-hSyn-DIO-mCherry by means of a calcium phosphate method. The purified virus particles were concentrated with an Amicon 10K MWCO filter (Merck Millipore), and the copy number of the viral genome was determined by the use of PCR. In some experiments, the same vector purchased from Addgene (50459-AAV5; https://www.addgene.org/50459/) was used.

### Stereotactic surgery

For stereotactic surgery, adult male mice (11–24 weeks old) were anesthetized with a mixture of midazolam, medetomidine, and butorphanol (4, 0.75, and 5 mg/kg body weight, respectively) and head-fixed on a stereotaxic frame (David Kopf Instruments). To label D1-MSNs and D2-MSNs, AAV5-hSyn-DIO-mCherry (1.1 × 10^13^ viral genome/ml, 0.5 μl) was injected into the bilateral NAc shell of the *Drd1*-*Cre* and *Drd2*-*Cre* mice, respectively, at a rate of 200 nl/min through a burr hole by the use of a pressure microinjector (KDS 101; KD Scientific). The stereotactic coordinates for the NAc shell (in mm, relative to the bregma) were AP 1.5, ML ±0.5, DV 4.5. The injection needle was kept in place for 5 min and then slowly retracted. Two to thirteen weeks after the virus injection, the mice were subjected to electrophysiological recordings. To study juvenile mice, male mice were injected at Postnatal Day (P)20 and analyzed at P28–34 by means of the same above-described method used in the adult mice.

### Slice preparation

Brain slices were obtained from 17- to 27-week-old mice as reported previously (Suzuki and Momiyama, 2021; Nishijo et al., 2022). For juvenile mice, brain slices were obtained at P28–34 (4–5 weeks). Briefly, after the mice had been decapitated under deep isoflurane anesthesia and their brains quickly dissected, coronal brain slices (300 μm thick) were cut by means of a microslicer (LinearSlicer PRO-7; Dosaka) in an ice-cold oxygenated modified cutting solution with the following composition (in mM): 92 choline chloride; 2.5 KCl; 30 NaHCO_3_; 1.2 NaH_2_PO_4_; 20 HEPES; 25 d-glucose; 5 ascorbic acid; 2 thiourea; 3 sodium pyruvate; 12 *N*-acetyl-l-cysteine; 0.5 CaCl_2_; and 10 MgCl_2_, pH 7.2, adjusted with *N*-methyl-d(-)-glucamine. The slices containing the NAc region were transferred to a recovery chamber containing the modified cutting solution at 32–34°C for 10 min and then incubated at room temperature in a holding chamber containing standard Krebs’ solution with the following composition (in mM): 124 NaCl; 3 KCl; 26 NaHCO_3_;, 1 NaH_2_PO_4_; 2.4 CaCl_2_; 1.2 MgCl_2_; and 10 d-glucose, pH 7.4, when bubbled with 95% O_2_ and 5% CO_2_.

### Whole-cell patch–clamp recording

The electrophysiological experiment data in each section were obtained from 7 to 14 neurons of 3–6 adult mice. For the juvenile mice, data in each strain were obtained from 12 to 13 neurons of three mice. The numbers of animals and cells used for each experiment are described in the corresponding figure legends or text. For recording, a brain slice was transferred to a recording chamber, held submerged, and superfused with standard Krebs’ solution (bubbled with 95% O_2_ and 5% CO_2_, 32–34°C) at a rate of 2–3 ml/min. The mCherry-positive cells in the NAc shell region were identified by means of an appropriate fluorescence filter (U-MWIG3; Olympus) and a 40× water immersion objective lens attached to an upright microscope (BX51WI; Olympus). Images were detected with a CCD camera (IR-1000; DAGE-MTI) and displayed on a video monitor (VU-17; DAGE-MTI). Considering the potential cytotoxicity associated with high levels of mCherry expression, cells with low to moderate fluorescence intensity were selected for recording. We performed whole-cell patch–clamp recordings from D1- and D2-MSNs in the NAc shell using a patch-clamp amplifier (MultiClamp 700B; Molecular Devices). Patch electrodes were pulled from Standard Wall Borosilicate Glass with Filament (1.5 mm outer diameter; Sutter Instrument) by means of a pipette puller (P-97; Sutter Instrument). They had resistances of 3–6 MΩ when filled with the internal solution. Data were low-pass filtered at 5 kHz and stored at 10–20 kHz. We used the pCLAMP system for data acquisition and Igor Pro 8 (WaveMetrics) with NeuroMatic (Rothman and Silver, 2018) and OriginPro2025 (OriginLab) for the analysis.

For current-clamp recording, patch pipettes were filled with a solution containing the following (in mM): 135 potassium gluconate; 6 NaCl; 10 KCl; 10 K-HEPES; 2 Mg-ATP; 0.3 Na2-GTP; and 0.1 K-EGTA, pH 7.3, adjusted with 1 M KOH. A liquid junction potential (LJP) of 15.5 mV was corrected offline after the experiments by means of pCLAMP software (version 10; Molecular Devices). For voltage-clamp recording, patch pipettes were filled with a solution containing the following (in mM): 140 CsCl; 9 NaCl; 1 Cs-EGTA; 10 Cs-HEPES; and 2 Mg-ATP, pH 7.3, adjusted with 1 M CsOH. The LJP was 5 mV, which was not compensated. The range of series resistance was 4.1–30 MΩ (11.18 ± 1.30 MΩ) in juvenile MSNs and 6.3–35 MΩ (17.50 ± 1.48 MΩ) in adult MSNs in the current-clamp mode and 4.87–28.3 MΩ (12.66 ± 0.89 MΩ) in adult MSNs in the voltage-clamp mode. These values were not compensated. Data were excluded from the analysis if the series resistance changed by >20% of the initial value.

Membrane resistance was obtained from the steady-state voltage response to a −200 pA current injection, with bridge balance compensation performed during current-clamp recordings. Increasing step currents from +20 to +440 pA in increments of 20 pA were injected for 500 ms to assess membrane potential responses under the current-clamp mode. The action potential amplitude was determined by measuring the difference between the peak membrane potential and the threshold potential. The rheobase was defined as the minimum depolarizing current required to evoke an action potential during step current injections. We performed phase plot analysis (Trombin et al., 2011) and determined the threshold for the action potential to be the point at which the membrane potential changed by >10 mV within 1 ms. Latency was determined by measuring the time from the start of current injection to the point at which the membrane potential reached the action potential threshold. The afterhyperpolarization (AHP) amplitude was defined as the difference between the threshold of the action potential and the peak of the hyperpolarization.

To evoke glutamatergic excitatory postsynaptic currents (EPSCs), we placed a glass pipette filled with 1 M NaCl 100–200 µm from the recorded neuron, and we delivered electrical stimulations every 5 s (100 μs duration, 0.2 Hz) in the presence of 10 μM bicuculline (Tocris Bioscience) and 0.5 μM strychnine (Sigma-Aldrich) to block GABA_A_ and glycine receptors, respectively. The stimulation intensity was adjusted to evoke EPSCs with an amplitude of approximately −100 and +100 pA at holding potentials of −80 and +40 mV, respectively. The AMPA current component of the evoked EPSCs was obtained by applying 25 μM d-AP5 (Tocris Bioscience) at a holding potential of +40 mV. The NMDA current was calculated by subtracting the AMPA current component from the EPSC recorded at +40 mV. The AMPA/NMDA ratio was then determined by dividing the peak amplitude of the AMPA current component by that of the NMDA current component. Bicuculline, strychnine, and d-AP5 were stored as frozen stock solutions and dissolved in the perfusing solution just before application in the final concentrations indicated.

### Statistical analysis

Data were analyzed by the use of Igor Pro 8, and statistical analyses were performed with OriginPro2025. For comparison of the electrophysiological parameters between the WT and *Sulf1* KO mice, statistical analyses were done with the nonparametric Mann–Whitney *U* test. To compare the data of multiple current stimulus intensities (Figs 2*C*, 3*B*,*G*, 4*B*,*G*), statistical analyses were done with two-way mixed–model ANOVA followed by the Bonferroni’s post hoc test. Statistical differences were considered significant at *p* < 0.05. All the statistical values are described in the corresponding figure legends or text and summarized in Table 1.

**Table 1.** Results from statistical analyses

Figure	Data structure	Type of test	Sample size	Statistical data
Figure 2				
Figure 2*A* Resting membrane potential (V rest) D1-MSN vs D2-MSN	Unknown	Mann–Whitney *U* test	D1-MSN, *n* = 10	*U* test, D1-MSN vs D2-MSN
			D2-MSN, *n* = 7	*U* = 8; *p* = 0.0097
Figure 2*B* Membrane resistance (R_m_) D1-MSN vs D2-MSN	Unknown	Mann–Whitney *U* test	D1-MSN, *n* = 10	*U* test, D1-MSN vs D2-MSN
			D2-MSN, *n* = 7	*U* = 30; *p* = 0.66
Figure 2*C* Number of action potentials D1-MSN vs D2-MSN	Normally distributed	Two-way mixed-model ANOVA followed by Bonferroni's multiple-comparison test	D1-MSN, *n* = 10	Cell type, *F*_(1,15)_ = 8.21; *p* = 0.012
			D2-MSN, *n* = 7	Current, *F*_(21,315)_ = 17.9; *p* < 0.0001
				Interaction, *F*_(21,315)_ = 9.05; *p* < 0.0001
				Multiple comparisons
				D1-MSN vs D2-MSN
				At 360 pA, *t* = 4.86; *p* = 0.0018
				At 380 pA, *t* = 5.60; *p* < 0.0001
				At 400 pA, *t* = 6.15; *p* < 0.0001
				At 420 pA, *t* = 6.64; *p* < 0.0001
				At 440 pA, *t* = 6.77; *p* < 0.0001
Figure 2*D* Rheobase D1-MSN vs D2-MSN	Unknown	Mann–Whitney *U* test	D1-MSN, *n* = 10	*U* test, D1-MSN vs D2-MSN
			D2-MSN, *n* = 7	*U* = 25.5; *p* = 0.38
Figure 2*E* Threshold of action potential D1-MSN vs D2-MSN	Unknown	Mann–Whitney *U* test	D1-MSN, *n* = 10	*U* test, D1-MSN vs D2-MSN
			D2-MSN, *n* = 7	*U* = 17; *p* = 0.088
Figure 2*F* AHP amplitude at rheobase D1-MSN vs D2-MSN	Unknown	Mann–Whitney *U* test	D1-MSN, *n* = 10	*U* test, D1-MSN vs D2-MSN
			D2-MSN, *n* = 7	*U* = 24; *p* = 0.31
Figure 3				
Figure 3*A* top Resting membrane potential (V rest) in D1-MSN WT vs KO	Unknown	Mann–Whitney *U* test	WT, *n* = 10	*U* test, WT vs KO
			KO, *n* = 11	*U* = 19; *p* = 0.012
Figure 3*A* bottom Membrane resistance (R_m_) in D1-MSN WT vs KO	Unknown	Mann–Whitney *U* test	WT, *n* = 10	*U* test, WT vs KO
			KO, *n* = 11	*U* = 27; *p* = 0.053
Figure 3*B* Number of action potentials in D1-MSN WT vs KO	Normally distributed	Two-way mixed–model ANOVA	WT, *n* = 10	Genotype, *F*_(1,19)_ = 0.41; *p* = 0.53
			KO, *n* = 11	Current, *F*_(21,399)_ = 8.51; *p* < 0.0001
				Interaction, *F*_(21,399)_ = 0.12; *p* = 1
Figure 3*C* AP amplitude in D1-MSN WT vs KO	Unknown	Mann–Whitney *U* test	WT, *n* = 10	*U* test, WT vs KO
			KO, *n* = 11	*U* = 37; *p* = 0.22
Figure 3*D* Rheobase in D1-MSN WT vs KO	Unknown	Mann–Whitney *U* test	WT, *n* = 10	*U* test, WT vs KO
			KO, *n* = 11	*U* = 48; *p* = 0.64
Figure 3*E* Latency at 440 pA current injection in D1-MSN WT vs KO	Unknown	Mann–Whitney *U* test	WT, *n* = 10	*U* test, WT vs KO
			KO, *n* = 11	*U* = 47; *p* = 0.60
Figure 3*F* Threshold of AP at 440 pA current injection in D1-MSN WT vs KO	Unknown	Mann–Whitney *U* test	WT, *n* = 10	*U* test, WT vs KO
			KO, *n* = 11	*U* = 54; *p* = 0.97
Figure 3*G* AHP amplitude at rheobase and 440 pA current injection in D1-MSN WT vs KO	Normally distributed	Two-way mixed–model ANOVA	WT, *n* = 10	Genotype, *F*_(1,19)_ = 0.75; *p* = 0.40
			KO, *n* = 11	AHP, *F*_(1,19)_ = 37.2; *p* < 0.0001
				Interaction, *F*_(1,19)_ = 0.000001; *p* = 0.999
Figure 4				
Figure 4*A* top Resting membrane potential (V rest) in D2-MSN WT vs KO	Unknown	Mann–Whitney *U* test	WT, *n* = 7	*U* test, WT vs KO
			KO, *n* = 14	*U* = 30; *p* = 0.17
Figure 4*A* bottom Membrane resistance (R_m_) in D2-MSN WT vs KO	Unknown	Mann–Whitney *U* test	WT, *n* = 7	*U* test, WT vs KO
			KO, *n* = 14	*U* = 37; *p* = 0.39
Figure 4*B* Number of action potentials in D2-MSN WT vs KO	Normally distributed	Two-way mixed–model ANOVA followed by Bonferroni's multiple-comparison test	WT, *n* = 7	Genotype, *F*_(1,19)_ = 1.01; *p* = 0.33
			KO, *n* = 14	Current, *F*_(21,399)_ = 26.2; *p* < 0.0001
				Interaction, *F*_(21,399)_ = 5.83; *p* = < 0.0001
				Multiple comparisons
				at 420 pA, *t* = 4.41; *p* = 0.013
				at 440 pA, *t* = 4.41; *p* = 0.013
Figure 4*C* AP amplitude in D2-MSN WT vs KO	Unknown	Mann–Whitney *U* test	WT, *n* = 7	*U* test, WT vs KO
			KO, *n* = 14	*U* = 22; *p* = 0.048
Figure 4*D* Rheobase in D2-MSN WT vs KO	Unknown	Mann–Whitney *U* test	WT, *n* = 7	*U* test, WT vs KO
			KO, *n* = 14	*U* = 17; *p* = 0.011
Figure 4*E* Latency at 440 pA current injection in D2-MSN WT vs KO	Unknown	Mann–Whitney *U* test	WT, *n* = 7	*U* test, WT vs KO
			KO, *n* = 14	*U* = 32.5; *p* = 0.23
Figure 4*F* Threshold of AP at 440 pA current injection in D2-MSN WT vs KO	Unknown	Mann–Whitney *U* test	WT, *n* = 7	*U* test, WT vs KO
			KO, *n* = 14	*U* = 42; *p* = 0.63
Figure 4*G* AHP amplitude at rheobase and 440 pA current injection in D2-MSN WT vs KO	Normally distributed	Two-way mixed–model ANOVA followed by Bonferroni's multiple-comparison test	WT, *n* = 7	Genotype, *F*_(1,19)_ = 0.14; *p* = 0.71
			KO, *n* = 14	AHP, *F*_(1,19)_ = 39.2; *p* < 0.0001
				Interaction, *F*_(1,19)_ = 17.9; *p* = 0.00046
				Multiple comparisons
				Rheobase AHP vs 440 pA AHP
				Within WT, *t* = 1.03; *p* = 1
				Within KO, *t* = 7.52; *p* < 0.0001
Figure 5				
Figure 5*A*, right AMPA/NMDA ratio in D1-MSN WT vs KO	Unknown	Mann–Whitney *U* test	WT, *n* = 10	*U* test, WT vs KO
			KO, *n* = 11	*U* = 10; *p* = 0.0017
Figure 5*B*, right AMPA/NMDA ratio in D2-MSN WT vs KO	Unknown	Mann–Whitney *U* test	WT, *n* = 10	*U* test, WT vs KO
			KO, *n* = 11	*U* = 27; *p* = 0.053

## Results

### *Sulf1* expression in the NAc shell

**Figure 1.**
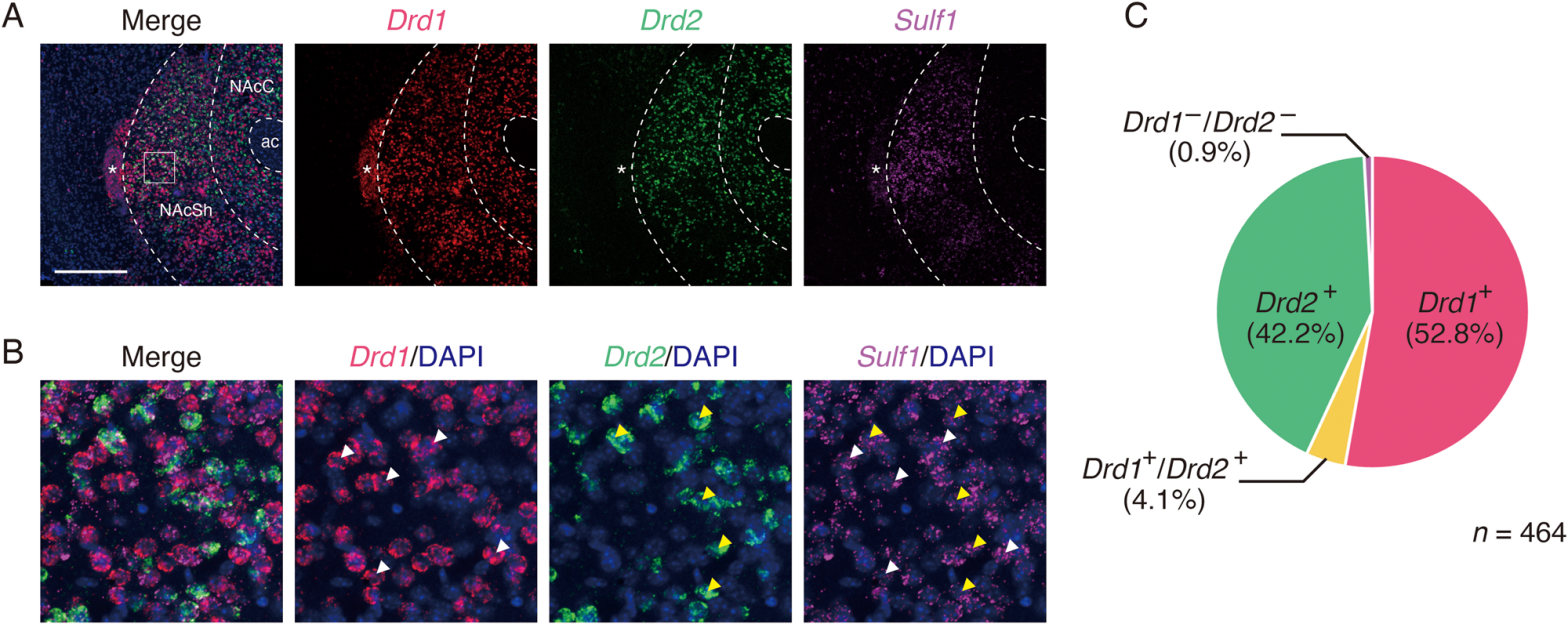
Expression of *Sulf1* mRNA in D1- and D2-MSNs. ***A***, Representative images of triple fluorescence in situ hybridization for *Drd1* (red), *Drd2* (green), and *Sulf1* (magenta). Broken lines indicate the border of the NAc shell. Asterisks indicate the major island of Calleja. ac, anterior commissure; NAcC, nucleus accumbens core; NAcSh, nucleus accumbens shell. ***B***, Magnified images of the boxed region in ***A***. White and yellow arrowheads indicate the representative cells coexpressing *Sulf1* and *Drd1*, and *Sulf1* and *Drd2*, respectively. ***C***, Percentages of *Drd1/Drd2*-expressing cells in the *Sulf1*-expressing cells in the NAc shell. The pie chart shows percentages of *Drd1* single-positive (*Drd1*^+^, red), *Drd2* single-positive (*Drd2*^+^, green), *Drd1/Drd2* double-positive (*Drd1*^+^/*Drd2*^+^, yellow), and *Drd1/Drd2* double-negative (*Drd1*^−^/*Drd2*^−^, dark magenta) cells in the *Sulf1*-expressing cells (*n* = 464, 6 slices from 3 mice). Scale bars, ***A***, 400 μm; ***B***, 50 μm.

We previously reported that *Sulf1* mRNA is highly expressed in the NAc of the adult mouse brain (Miya et al., 2021). We also showed coexpression of *Sulf1* and dopamine D1 and D2 receptors by the use of combination of immunohistochemistry and AAV vector-mediated labeling of D1- and D2-MSNs. These experiments revealed that *Sulf1* is expressed abundantly in most of the D1- and D2-MSNs in the NAc shell (Miya et al., 2021). In this study, to directly confirm the overlapping expression of *Sulf1*, *Drd1*, and *Drd2* mRNAs, we performed multiplex fluorescence in situ hybridization. When observed at low magnification, strong signals for *Drd1* and *Drd2* were observed throughout the NAc and the *Sulf1* signals mainly in the NAc shell, with higher density in its dorsomedial portion (Fig. 1*A*). At high magnification, both the *Drd1* and the *Drd2* signals overlapped with the *Sulf1* signals (Fig. 1*B*). The vast majority of *Drd1*^+^ and *Drd2*^+^ cells coexpressed *Sulf1*: 98.9% of *Drd1*^+^ cells (*n* = 264) and 99.5% of *Drd2*^+^ cells (*n* = 215) were positive for *Sulf1*. Among the *Sulf1*-expressing cells, when *Drd1/Drd2* double-positive cells were included, 56.9% were positive for *Drd1* and 46.3% for *Drd2* (Fig. 1*C*). These data were close to the reported fractions of D1-MSNs and D2-MSNs in the NAc shell (Bertran-Gonzalez et al., 2008; Kupchik et al., 2015; Miyasaka et al., 2025). About 4% were positive for both *Drd1* and *Drd2*, similar to the percentages reported previously (Kupchik et al., 2015; Miyasaka et al., 2025). These results indicate that almost all the MSNs in the NAc shell express *Sulf1*.

### Differences of electrophysiological properties in D1-MSNs and D2-MSNs

To study the roles of *Sulf1* in the NAc, we decided to examine the electrophysiological properties of MSNs by means of whole-cell patch–clamp recording. Considering the possibility that *Sulf1* KO may have different effects on D1-MSNs and D2-MSNs, we adopted a strategy of recording them separately. To this end, we labeled D1-MSNs and D2-MSNs separately by stereotactic injection of a Cre-dependent AAV vector expressing a red fluorescent protein, mCherry, into *Drd1-Cre* and *Drd2-Cre* mice homozygous for the WT or KO allele of the *Sulf1* gene. Two to thirteen weeks after virus injection, we identified mCherry-positive cells in the medial shell region of the NAc in 300 μm-thick slices and performed whole-cell patch–clamp recordings.

**Figure 2.**
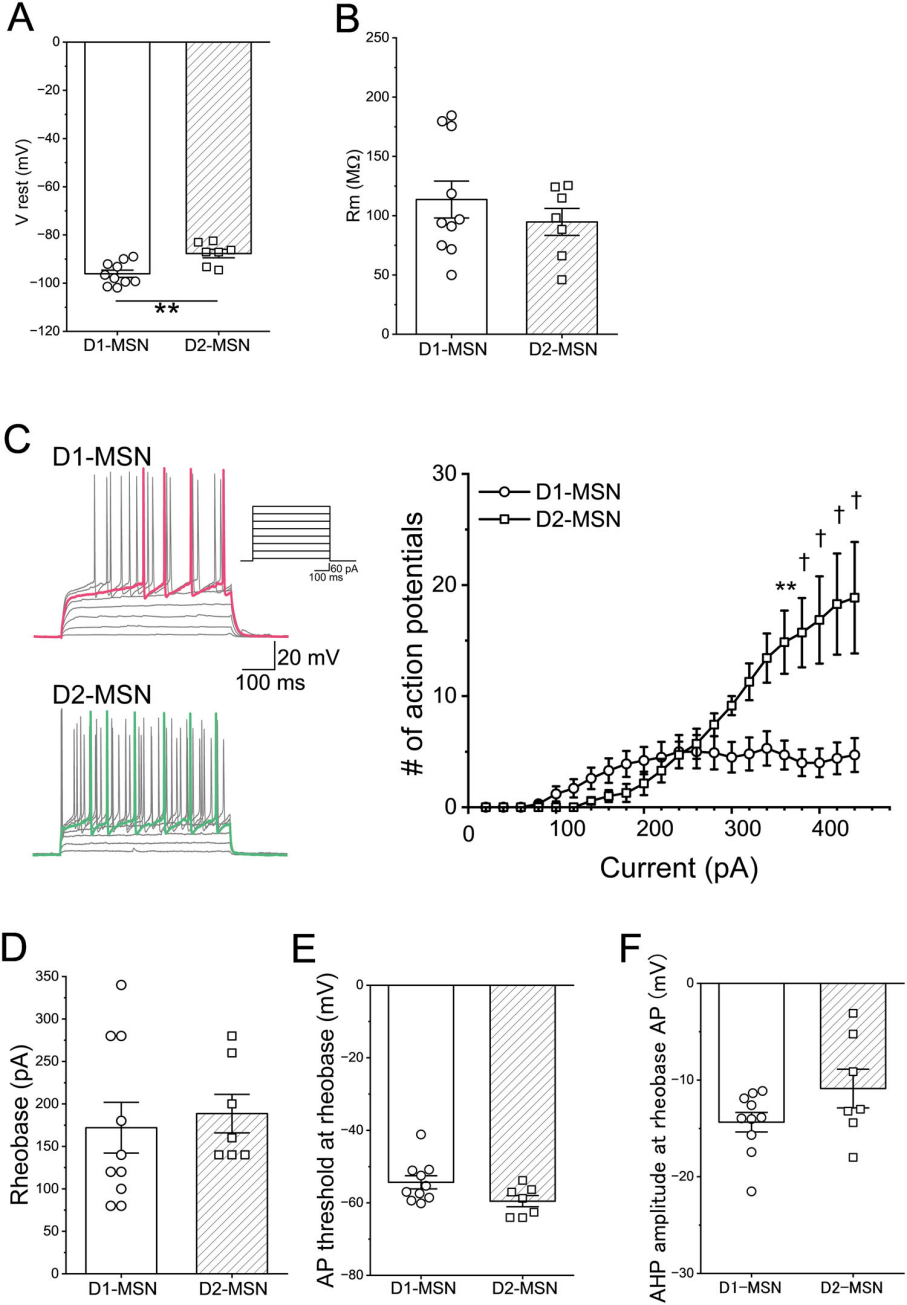
Comparison of electrophysiological properties between D1-MSNs and D2-MSNs of the WT mice. ***A***, Resting membrane potential (V rest). The V rest in the D1-MSNs (−96.09 ± 1.49 mV, *n* = 10 neurons from 4 mice) was significantly more hyperpolarized than that in the D2-MSNs (−87.70 ± 1.76 mV, 7 neurons from 4 mice; *U* = 8; *p* = 0.0097; Mann–Whitney *U* test). Open circles and squares indicate D1-MSNs and D2-MSNs, respectively (same as below). ***B***, Membrane resistance (R_m_). The R_m_ were 113.64 ± 15.54 MΩ in the D1-MSNs (*n* = 10 neurons from 4 mice) and 94.71 ± 11.41 MΩ in the D2-MSNs (*n* = 7 neurons from 4 mice). The R_m_ did not differ significantly between the groups (*U* = 30; *p* = 0.66; Mann–Whitney *U* test). ***C***, Left, action potentials of D1-MSNs (top) and D2-MSNs (bottom) in WT mice. Membrane potential changes were elicited by depolarizing currents (+20 to +440 pA in 20 pA increments, 500 ms duration). Scale bars, 100 ms and 20 mV. The traces when action potentials were generated at a minimum injection current are shown in color (magenta in D1-MSNs and green in D2-MSNs). The top-right panel shows a part of the current injection protocol from +20 to +440 pA in 60 pA increments (scale bars, 100 ms and 60 pA). Right, relationship between injected currents and number of action potentials. At 360–440 pA current injections, the numbers of action potentials were significantly higher in the D2-MSNs (*n* = 7 neurons from 4 mice) than in the D1-MSNs (10 neurons from 4 mice; main effect of cell type, *F*_(1,15)_ = 8.21; *p* = 0.012; main effect of current, *F*_(21,315)_ = 17.9; *p* < 0.0001; interaction; *F*_(21,315)_ = 9.05; *p* < 0.0001; Bonferroni’s post hoc test, at 360 pA, *t* = 4.86; *p* = 0.0018; at 380 pA; *t* = 5.60; *p* < 0.0001; at 400 pA, *t* = 6.15; *p* < 0.0001; at 420 pA, *t* = 6.64; *p* < 0.0001; and at 440 pA, *t* = 6.77; *p* < 0.0001; two-way mixed–model ANOVA). ***D***, Rheobase in D1-MSNs and D2-MSNs. The rheobases were 172.00 ± 29.84 pA in the D1-MSNs (*n* = 10 neurons from 4 mice) and 188.57 ± 22.62 pA in the D2-MSNs (*n* = 7 neurons from 4 mice). They did not differ significantly between the groups (*U* = 25.5; *p* = 0.38; Mann–Whitney *U* test). ***E***, Threshold of the first action potential elicited by current injection. The thresholds were −54.35 ± 1.81 mV in the D1-MSNs (*n* = 10 neurons from 4 mice) and −59.54 ± 1.54 mV in the D2-MSNs (*n* = 7 neurons from 4 mice). They did not differ significantly between the groups (*U* = 17; *p* = 0.088; Mann–Whitney *U* test). ***F***, Amplitude of AHP of the first action potential elicited by the current injection. The AHP amplitudes were −14.36 ± 1.01 mV in the D1-MSNs (*n* = 10 neurons from 4 mice) and −10.88 ± 2.00 mV in the D2-MSNs (*n* = 7 neurons from 4 mice). They did not differ significantly between the groups (*U* = 24; *p* = 0.31; Mann–Whitney *U* test). These data are a reanalysis of the WT mouse data presented in Figures 3 and 4. Data are presented as means ± SEMs. **p < 0.01; ^†^*p* < 0.0001. Extended Data Figure 2-1 shows comparison of electrophysiological properties between D1-MSNs and D2-MSNs of juvenile WT mice. Extended Data Figure 2-2 shows the results of statistical analyses of juvenile mouse studies in Extended Data Figure 2-1.

Before starting the analysis of *Sulf1* KO mice, we compared the membrane properties of D1-MSNs and D2-MSNs in the adult WT mice because several studies have reported electrophysiological differences between D1-MSNs and D2-MSNs in the dorsal striatum (Ma et al., 2012; Schier et al., 2017; Willett et al., 2019) and NAc (Francis et al., 2015; Al-Muhtasib et al., 2018; Cao et al., 2018). When the resting membrane potentials were measured under the current-clamp mode, the D1-MSNs were significantly more hyperpolarized than were the D2-MSNs, whereas the membrane resistances did not differ significantly between them (Fig. 2*A*,*B*). We next examined the neuronal firing responses to depolarizing current injections. The D1-MSNs showed a higher spike frequency than that of the D2-MSNs at low injected currents (100–240 pA, not statistically significant) but did not show an increase in the spike number at currents larger than 240 pA (Fig. 2*C*). In contrast, in the D2-MSNs, the spike numbers increased linearly as the current increased and the spike frequency was higher than in the D1-MSNs at large injected currents (Fig. 2*C*; D1-MSN, *n* = 10 neurons from four mice; D2-MSN, *n* = 7 neurons from four mice; main effect of the cell type, *F*_(1,15)_ = 8.21; *p* = 0.012; main effect of current, *F*_(21,315)_ = 17.9; *p* < 0.0001; interaction, *F*_(21,315)_ = 9.05; *p* < 0.0001; two-way mixed–model ANOVA). No differences were observed between the D1-MSNs and D2-MSNs in the rheobase, the threshold of the first action potential during current injection, and the AHP amplitude at the rheobase (Fig. 2*D*–*F*). These results suggest that D1-MSNs and D2-MSNs in the NAc shell of the adult WT mice have distinct membrane properties and excitabilities.

10.1523/ENEURO.0088-25.2025.f2-1 Figure 2-1 Comparison of electrophysiological properties between D1-MSNs and D2-MSNs of juvenile WT mice. ***A***, Resting membrane potential (V rest). The V rest did not differ between the D1-MSNs (−91.61 ± 1.21 mV, *n* = 12 neurons from 3 mice) and D2-MSNs (−90.30 ± 1.89 mV, 13 neurons from 3 mice; *U* = 71, *p* = 0.72; Mann-Whitney U test). Open circles and squares indicate D1-MSNs and D2-MSNs, respectively (same as below). ***B***, Membrane resistance (R_m_). The R_m_ did not differ between the D1-MSNs (140.73 ± 19.78 MΩ, *n* = 12 neurons from 3 mice) and D2-MSNs (105.58 ± 11.76 MΩ, *n* = 13 neurons from 3 mice; *U* = 52, *p* = 0.17; Mann-Whitney U test). ***C***, Left, action potentials of D1-MSNs (top) and D2-MSNs (bottom) in juvenile WT mice. Membrane potential changes were elicited by depolarizing currents (+20 to +440 pA in 20 pA increments, 500-ms duration). Scale bars, 100 ms and 20 mV. The traces when action potentials were generated at a minimum injection current are shown in color (magenta in D1-MSNs and green in D2-MSNs). The upper right panel shows a part of the current injection protocol from +20 pA to +440 pA in 60-pA increments (scale bars, 100 ms and 60 pA). Right, relationship between injected currents and number of action potentials. They did not differ significantly between the groups (main effect of cell type, *F*(1,23) = 0.42, *p* = 0.53; main effect of current, *F*(21,483) = 33.8, *p* < 0.0001; interaction; *F*(21,483) = 0.31, *p* = 0.999). Download Figure 2-1, TIF file.

10.1523/ENEURO.0088-25.2025.f2-2 Figure 2-2 Results of statistical analyses of juvenile mouse studies in Figure 2-1. Download Figure 2-2, DOCX file.

The excitability of D1-MSNs and D2-MSNs in the adult NAc shell described above differs significantly from those reported in the dorsal striatum (Ma et al., 2012; Schier et al., 2017; Willett et al., 2019) and NAc core (Al-Muhtasib et al., 2018; Cao et al., 2018): in previous reports, D1-MSNs were generally harder to excite than were D2-MSNs. Because those previous studies used juvenile mice, we considered that this might be due to differences in the age of the mice analyzed. We thus performed electrophysiological recording of the D1-MSNs and D2-MSNs in the NAc shell of the juvenile mice. For this purpose, we injected *Drd1-Cre* and *Drd2-Cre* mice with AAV-DIO-mCherry at P20 and performed slice patch-clamp recording at P28–34 by means of the same method used in the adult mice. As a result, we found no significant differences in the resting membrane potential and membrane resistance between the D1-MSNs and D2-MSNs (Extended Data Figs. 2-1*A*,*B*, 2-2). In addition, the numbers of action potentials after current injection did not differ significantly between the D1-MSNs and D2-MSNs (Extended Data Figs. 2-1*C*, 2-2).

### Effects of *Sulf1* disruption on the intrinsic electrophysiological properties and neuronal excitability of NAc D1-MSNs

**Figure 3.**
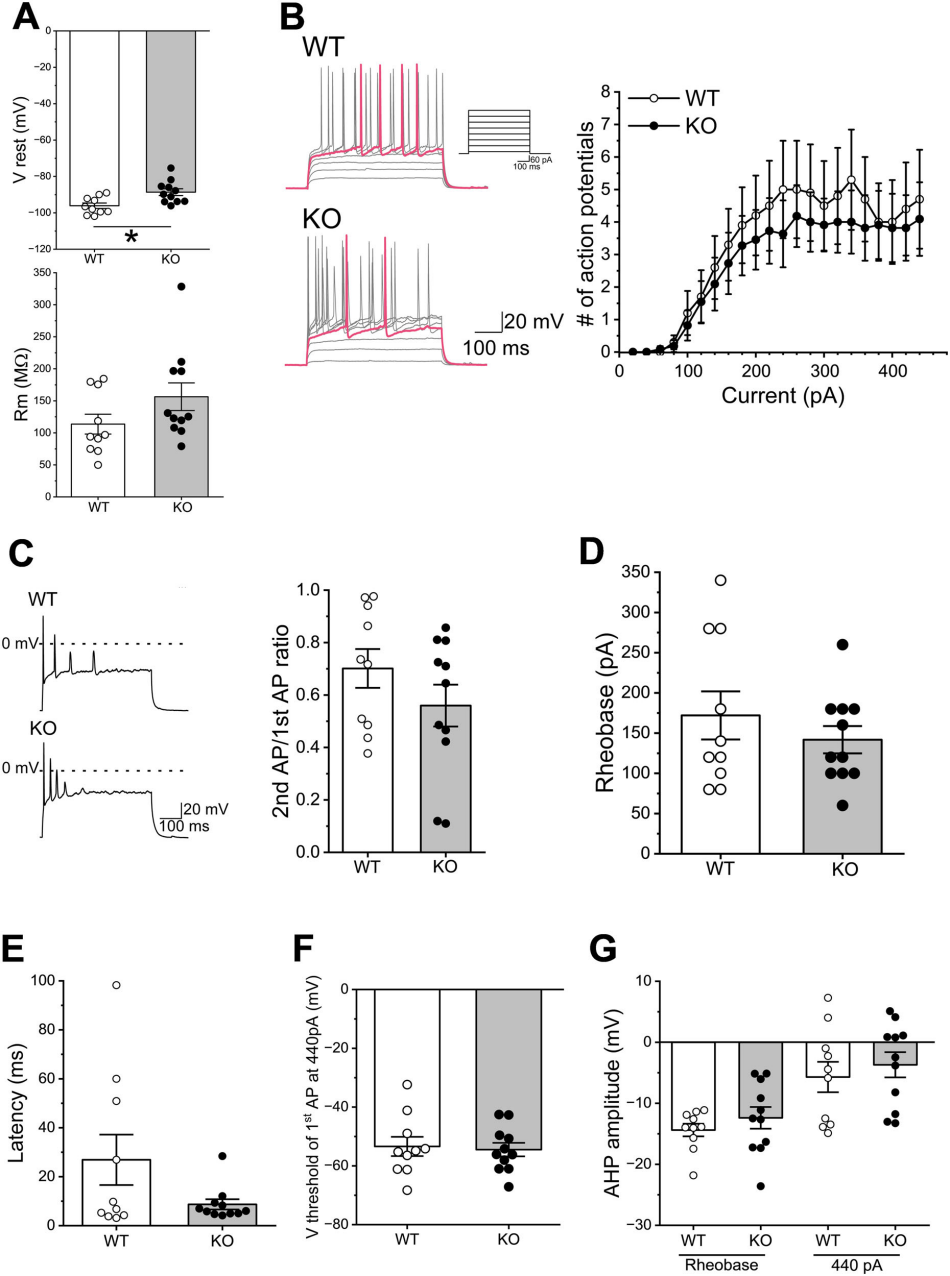
Electrophysiological properties and neuronal excitability of D1-MSNs in WT and *Sulf1* KO mice. ***A***, Top, resting membrane potential (V rest) of D1-MSNs. Open and closed circles indicate control WT and *Sulf1* KO mice, respectively (same as below). The V rest in the KO mice (−88.63 ± 1.87 mV, *n* = 11 neurons from 4 mice) was significantly more depolarized than that in the WT mice (−96.09 ± 1.49 mV, *n* = 10 neurons from 4 mice; *U* = 19; *p* = 0.012; Mann–Whitney *U* test). Bottom, membrane resistance (R_m_) of D1-MSNs. The R_m_ were 113.64 ± 15.54 MΩ in the WT mice (*n* = 10 neurons from 4 mice) and 156.40 ± 21.54 MΩ in the KO mice (*n* = 11 neurons from 4 mice). The R_m_ did not differ significantly between the groups (*U* = 27; *p* = 0.053; Mann–Whitney *U* test). ***B***, Left, action potentials of D1-MSNs of the WT (top) and KO (bottom) mice. Membrane potential changes were elicited by depolarizing currents (+20 to +440 pA in 20 pA increments, 500 ms duration). Scale bars, 100 ms and 20 mV. The traces when action potentials were generated at a minimum injection current are shown in magenta. The top-right panel shows a part of the current injection protocol from +20 to +440 pA in 60 pA increments (scale bars, 100 ms and 60 pA). Right, relationship between injected currents and number of action potentials. The number of action potentials did not differ between the WT and KO mice (main effect of genotype, *F*_(1,19)_ = 0.41; *p* = 0.53; main effect of current, *F*_(21,399)_ = 8.51; *p* < 0.0001, interaction, *F*_(21,399)_ = 0.12; *p* = 1). ***C***, Comparison of the first and second action potentials. Left, action potentials evoked by a 440 pA current injection (500 ms duration) in the WT (top) and KO (bottom) mice. Right, the ratio of the second and first action potential amplitudes. There was no significant difference in the reduction ratio between the groups (*U* = 37; *p* = 0.22; Mann–Whitney *U* test). ***D***, Rheobase in WT and KO mice. The rheobases were 172.00 ± 29.84 pA in the WT mice (*n* = 10 neurons from 4 mice) and 141.82 ± 16.94 pA in the KO mice (*n* = 11 neurons from 4 mice). They did not differ significantly between the groups (*U* = 48; *p* = 0.64; Mann–Whitney *U* test). ***E***, Latency of the first action potential evoked by a 440 pA current injection. The latencies were 26.90 ± 10.30 ms in the WT mice (*n* = 10 neurons from 4 mice) and 8.69 ± 2.10 ms in the KO mice (*n* = 11 neurons from 4 mice). They did not differ significantly between the groups (*U* = 47; *p* = 0.60; Mann–Whitney *U* test). ***F***, Threshold of the first action potential evoked by a 440 pA current injection. The thresholds were −53.40 ± 3.28 mV in the WT mice (*n* = 10 neurons from 4 mice) and −54.48 ± 2.30 mV in the KO mice (*n* = 11 neurons from 4 mice). They did not differ significantly between the groups (*U* = 54; *p* = 0.97; Mann–Whitney *U* test). ***G***, AHP amplitude of the first action potential at the rheobase and a 440 pA current injection. The AHP amplitudes at the rheobase were −14.39 ± 1.03 mV in the WT mice (*n* = 10 neurons from 4 mice) and −12.39 ± 1.77 mV in the KO mice (*n* = 11 neurons from 4 mice). The AHP amplitudes at a 440 pA current injection were −5.70 ± 2.49 mV in the WT mice (*n* = 10 neurons from 4 mice) and −3.69 ± 2.06 mV in the KO mice (*n* = 11 neurons from 4 mice). The AHP amplitudes of the first action potentials evoked by a 440 pA current injection were smaller than those at the rheobase (main effect of genotype, *F*_(1,19)_ = 0.75; *p* = 0.40; main effect of AHP, *F*_(1,19)_ = 37.2,; *p* < 0.0001; interaction, *F*_(1,19)_ = 0.000001; *p* = 0.999; two-way mixed–model ANOVA). *p < 0.05.

Next, we compared the intrinsic electrophysiological properties of D1-MSNs between the adult WT and *Sulf1* KO mice. The resting membrane potentials were significantly more depolarized in the *Sulf1* KO mice than in the WT mice (Fig. 3*A*). No difference in the membrane resistance was observed between the WT and the *Sulf1* KO mice (Fig. 3*A*). We then examined the effects of *Sulf1* KO on the neuronal excitability of D1-MSNs. The number of action potentials in the D1-MSNs to depolarizing current injections from +20 to +440 pA did not differ significantly between the WT and the *Sulf1* KO mice (Fig. 3*B*; WT, *n* = 10 neurons from four mice; KO, *n* = 11 neurons form four mice; main effect of genotype, *F*_(1,19)_ = 0.41; *p* = 0.53; main effect of current, *F*_(21,399)_ = 8.51; *p* < 0.0001; interaction, *F*_(21,399)_ = 0.12; *p* = 1; two-way mixed-model ANOVA). However, we noticed that in some D1-MSNs, the action potentials evoked by large current injections exhibited attenuation of the amplitude, and it seemed that the tendency was pronounced in the KO mice. To investigate this point in detail, we compared the amplitudes of the first and second action potentials evoked by a 440 pA current injection in the WT and KO mice. In both genotypes, the amplitude of the second action potentials was lower than that of the first action potentials, and there was no significant difference in the reduction ratio between the genotypes (Fig. 3*C*; *U* = 37; *p* = 0.22; Mann–Whitney *U* test). No significant differences were observed between the WT and the KO mice in the rheobase (Fig. 3*D*) nor in the latency and threshold of the first action potential evoked by a 440 pA current injection (Fig. 3*E*,*F*). We next compared the AHP amplitude of the first action potentials at the rheobase and at a 440 pA current injection. In both the WT and the KO mice, the AHP amplitude of the first action potentials at the 440 pA current injection was smaller than that at the rheobase (Fig. 3*G*; main effect of genotype, *F*_(1,19)_ = 0.75; *p* = 0.40; main effect of AHP, *F*_(1,19)_ = 37.2; *p* < 0.0001; interaction, *F*_(1,19)_ = 0.000001; *p* = 0.999; two-way mixed–model ANOVA). These results suggest that *Sulf1* disruption resulted in changes in the resting membrane potential of the D1-MSNs.

### Effects of *Sulf1* disruption on the intrinsic electrophysiological properties and neuronal excitability of NAc D2-MSNs

**Figure 4.**
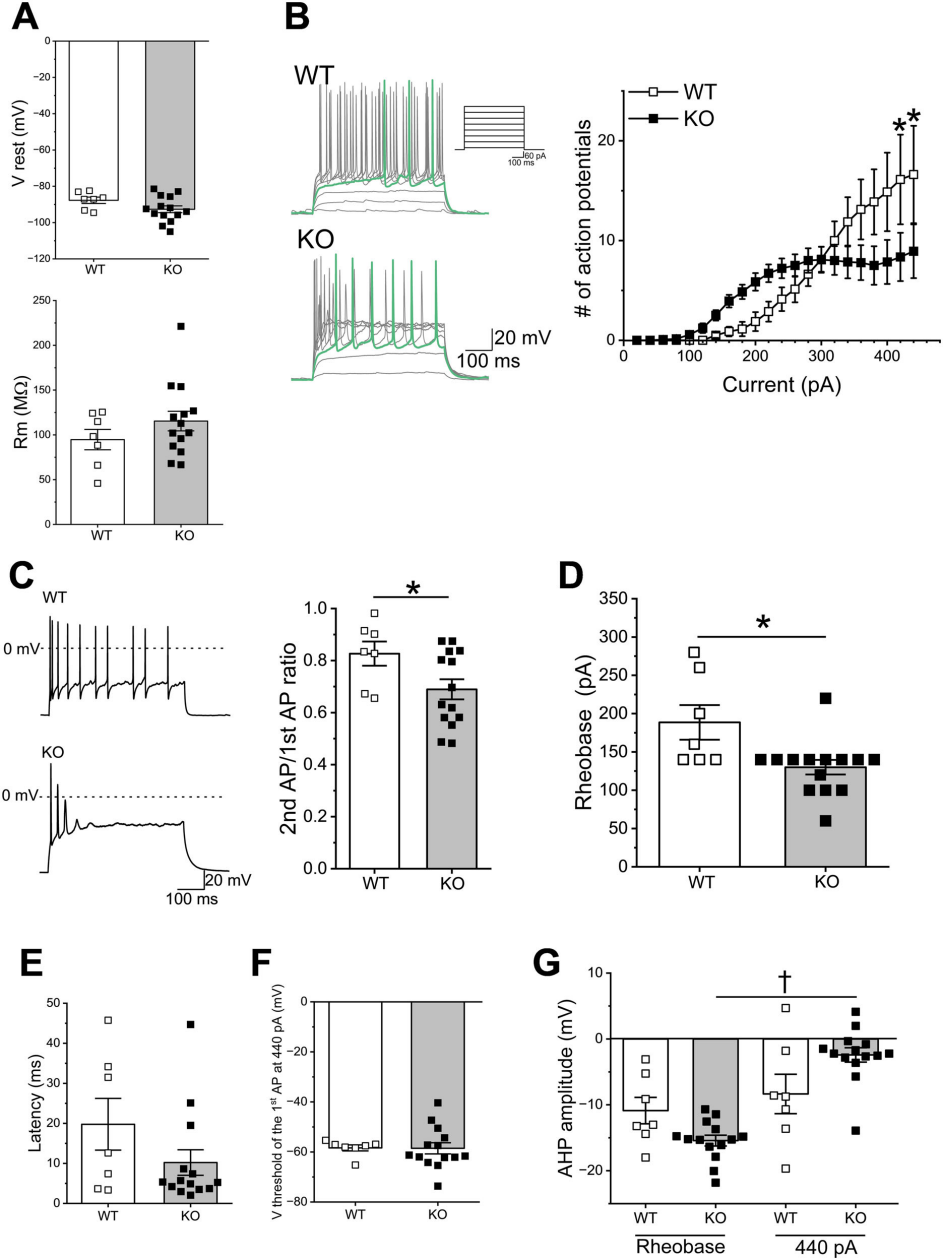
Electrophysiological properties and neuronal excitability of D2-MSNs in WT and *Sulf1* KO mice. ***A***, Top, resting membrane potentials (V rest) of D2-MSNs. The open and closed squares indicate control WT and *Sulf1* KO mice, respectively (same as below). The V rests were −87.70 ± 1.76 mV in the WT mice (*n* = 7 neurons from 4 mice) and −92.65 ± 1.88 mV in the KO mice (*n* = 14 neurons from 5 mice). They did not differ significantly between the groups (*U* = 30; *p* = 0.17; Mann–Whitney *U* test). Bottom, membrane resistances (R_m_) of D2-MSNs. The R_m_ were 94.7 ± 11.41 MΩ in the WT mice (*n* = 7 neurons from 4 mice) and 115.43 ± 10.93 MΩ in the KO mice (*n* = 14 neurons from 5 mice). They did not differ significantly different between the groups (*U* = 37; *p* = 0.39; Mann–Whitney *U* test). ***B***, Left, action potentials of D2-MSNs in WT (top) and KO (bottom) mice. Membrane potential changes were elicited by depolarizing currents (+20 to +440 pA in 20 pA increments, 500 ms duration). Scale bars, 100 ms and 20 mV. The traces when action potentials were generated at a minimum injection current are shown in green. The top-right panel shows a part of the current injection protocol from +20 to +440 pA in 60 pA increments (scale bars, 100 ms and 60 pA). Right, relationship between injected currents and number of action potentials. At 420 and 440 pA current injections, the numbers of action potentials were significantly higher in the WT mice (*n* = 7 neurons form 4 mice) than those in the KO mice (*n* = 14 neurons form 5 mice; main effect of genotype, *F*_(1,19)_ = 1.01; *p* = 0.33; main effect of current, *F*_(21,399)_ = 26.2; *p* < 0.0001; interaction, *F*_(21,399)_ = 5.83; *p* < 0.0001; Bonferroni’s post hoc test, at 420 pA, *t* = 4.41; *p* = 0.013; at 440 pA, *t* = 4.41; *p* = 0.013; two-way mixed–model ANOVA). ***C***, Comparison of the first and second action potentials. Left, action potentials evoked by a 440 pA current injection (500 ms duration) in the WT (top) and KO (bottom) mice. Right, The ratio of the second and first action potentials. The reduction ratio was significantly higher in the KO mice than in the WT mice (*U* = 22; *p* = 0.048; Mann–Whitney *U* test). ***D***, Rheobase in WT and KO mice. The rheobase in the KO mice (130.00 ± 9.55 pA; *n* = 14 neurons from 5 mice) was significantly smaller than that in the WT mice (188.57 ± 22.62 pA; *n* = 7 neurons from 4 mice; *U* = 17, *p* = 0.011; Mann–Whitney *U* test). ***E***, Latency of the first action potential evoked by a 440 pA current injection. The latencies were 19.77 ± 6.46 ms in the WT mice (*n* = 7 neurons from 4 mice) and 10.21 ± 3.19 ms in the KO mice (*n* = 14 neurons from 5 mice). They did not differ significantly between the groups (*U* = 32.5; *p* = 0.23; Mann–Whitney *U* test). ***F***, Threshold of the first action potential evoked by a 440 pA current injection. The thresholds were −58.37 ± 1.20 mV in the WT mice (*n* = 7 neurons from 4 mice) and −58.54 ± 2.23 mV in the KO mice (*n* = 14 neurons from 5 mice). They did not differ significantly between the groups (*U* = 42; *p* = 0.63; Mann–Whitney *U* test). ***G***, AHP amplitude of the first action potential evoked at the rheobase and 440 pA current injection. The AHP amplitudes at the rheobase were −10.88 ± 2.00 mV in the WT mice (*n* = 7 neurons from 4 mice) and −15.43 ± 0.81 mV in the KO mice (*n* = 14 neurons from 5 mice). The AHP amplitudes of the first action potential evoked by a 440 pA current injection were −8.35 ± 2.99 mV in the WT mice (*n* = 7 neurons from 4 mice) and −2.43 ± 1.09 mV in the KO mice (*n* = 14 neurons from 5 mice). The AHP amplitude of the first action potential at a 440 pA current injection was significantly smaller than that at the rheobase in the KO mice but not in the WT mice (main effect of genotype, *F*_(1,19)_ = 0.14; *p* = 0.71; main effect of AHP, *F*_(1,19)_ = 39.2; *p* < 0.0001; interaction; *F*_(1,19)_ = 17.9; *p* = 0.00046; Bonferroni’s post hoc test, AHP within WT, *t* = 1.03; *p* = 1; AHP within KO, *t* = 7.52; *p* < 0.0001; two-way mixed–model ANOVA). Data are presented as means ± SEMs. *p < 0.05; ^†^*p* < 0.0001.

Next, we compared the intrinsic electrophysiological properties of the D2-MSNs of the adult WT and the *Sulf1* KO mice. The D2-MSNs showed no significant differences in resting membrane potentials and membrane resistances between the WT and the *Sulf1* KO mice (Fig. 4*A*). We then analyzed the effects of *Sulf1* disruption on the excitability of D2-MSNs. When the membrane potential responses to depolarizing current injections (from +20 to +440 pA) were measured, marked differences were observed in the firing patterns between the WT mice and the *Sulf1* KO mice (Fig. 4*B*). In the *Sulf1* KO mice, the action potentials occurred at lower currents and were more frequent than in the WT mice between currents of 100 and 280 pA (Fig. 4*B*, not statistically significant). However, at a higher current (>300 pA), the number of action potentials did not increase in the *Sulf1* KO mice, whereas that in the WT mice increased as the stimulus currents became larger (Fig. 4*B*). These differences between the WT mice and the *Sulf1* KO mice were statistically significant (Fig. 4*B*; WT, *n* = 7 neurons from four mice; KO, *n* = 14 neurons from five mice; *F*_(1,19)_ = 1.01; *p* = 0.33; main effect of current, *F*_(21,399)_ = 26.2; *p* < 0.0001; interaction, *F*_(21,399)_ = 5.83; *p* < 0.0001; two-way mixed–model ANOVA). The Bonferroni’s post hoc analysis revealed that the D2-MSNs in the WT mice generated more spikes than those in the *Sulf1* KO mice at 420 and 440 pA current injections (Fig. 4*B*; at 420 pA; *t* = 4.41; *p* = 0.013, at 440 pA; *t* = 4.41; *p* = 0.013). We then compared the amplitudes of the first and second action potentials evoked by a 440 pA current injection (Fig. 4*C*). In both the WT and the *Sulf1* KO mice, the amplitudes of the second action potentials were smaller than those of the first action potentials, and the reduction ratio was significantly higher in the KO mice than in the WT mice (*U* = 22; *p* = 0.048; Mann–Whitney *U* test). The rheobase in the D2-MSNs of the *Sulf1* KO mice was significantly smaller than that in the WT mice (Fig. 4*D*). No differences were observed between the genotypes in the latency and threshold of the first action potential evoked by a 440 pA current injection (Fig. 4*E*,*F*). We next compared the AHP amplitude of the first action potentials at the rheobase and at a 440 pA current injection. In both the WT and the KO mice, the AHP amplitudes of the first action potentials at a 440 pA current injection appeared to be smaller than those at the rheobase (Fig. 4*G*; main effect of genotype, *F*_(1,19)_ = 0.14; *p* = 0.71; main effect of AHP, *F*_(1,19)_ = 39.2; *p* < 0.0001; interaction, *F*_(1,19)_ = 17.9; *p* = 0.00046; two-way mixed–model ANOVA). However, the Bonferroni’s post hoc test revealed that the degree of the decrease was significant in the KO mice but not in the WT mice (Fig. 4*G*; WT, *t* = 1.03; *p* = 1; KO, *t* = 7.52; *p* < 0.0001). These results suggest that the D2-MSNs of *Sulf1* KO mice are more easily activated by weak stimuli but tend to be inactivated by strong excitations.

### Glutamatergic synaptic transmission in *Sulf1* KO mice

We finally examined the effects of *Sulf1* disruption on excitatory synaptic transmission onto MSNs in the NAc of adult mice. The cells were voltage clamped at −80 mV, and glutamatergic EPSCs were evoked by focal electrical stimulation at 0.2 Hz in the presence of bicuculline (10 µM) and strychnine (0.5 µM). The EPSC amplitudes in the D1-MSNs were −137.59 ± 33.19 pA in the WT mice (*n* = 10 neurons from three mice) and −158.50 ± 30.29 pA in the KO mice (*n* = 11 neurons from six mice), and those in the D2-MSNs were −119.33 ± 16.61 in the WT mice (*n* = 10 neurons from four mice) and −218.21 ± 28.43 pA in the KO mice (*n* = 11 neurons from six mice). These data showed that normal glutamatergic transmission was maintained in the *Sulf1* KO mice.

**Figure 5.**
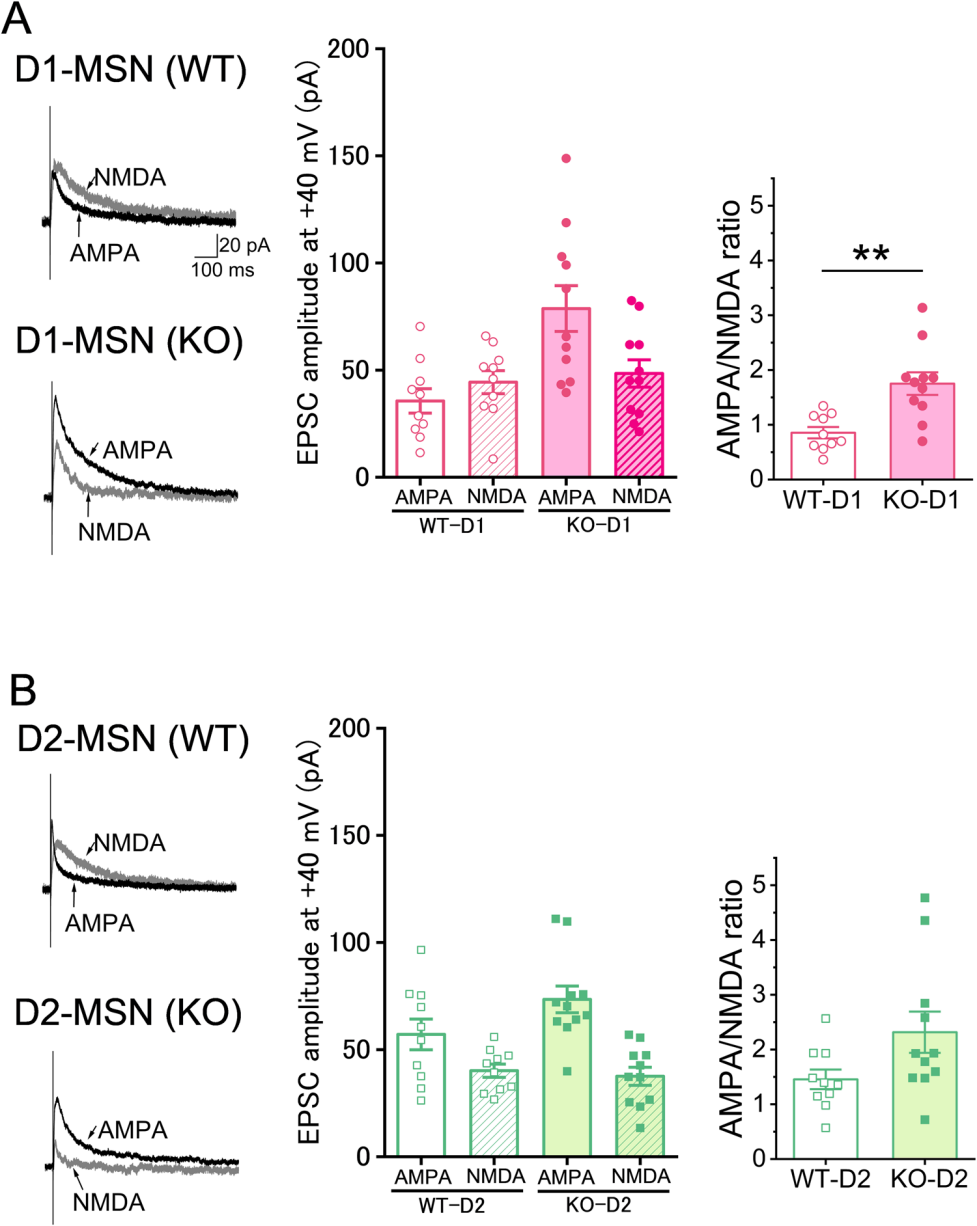
Glutamatergic transmission in D1-MSNs and D2-MSNs of WT and *Sulf1* KO mice. ***A***, Left, AMPA and NMDA EPSCs recorded under a voltage clamp at +40 mV in D1-MSNs. Scale bars, 100 ms and 20 pA. Middle, amplitude of AMPA EPSCs and NMDA EPSCs in D1-MSNs. The amplitudes of AMPA and NMDA EPSCs in the WT mice (*n* = 10 neurons from 3 mice) were 35.62 ± 5.71 pA and 44.37 ± 5.40 pA, respectively. The amplitudes of AMPA and NMDA EPSCs in the KO mice (*n* = 11 neurons from 5 mice) were 78.72 ± 10.72 pA and 48.44 ± 6.36 pA, respectively. Statistical analyses were not performed on the amplitudes of AMPA and NMDA EPSCs because these responses were elicited by stimuli of different sizes for each cell to obtain ∼100 pA responses (same as in ***B***). Right, The AMPA/NMDA ratio. The AMPA/NMDA ratio in the D1-MSNs of the KO mice (1.75 ± 0.21; *n* = 11 neurons from 5 mice) was significantly higher than that in the WT mice (0.85 ± 0.10; *n* = 10 neurons from 3 mice; *U* = 10; *p* = 0.0017; Mann–Whitney *U* test). ***B***, Left, AMPA and NMDA EPSCs recorded under a voltage clamp at +40 mV in D2-MSNs. Scale bars, 100 ms and 20 pA. Middle, Amplitude of AMPA and NMDA EPSCs in D2-MSNs. The amplitudes of AMPA and NMDA EPSCs in the WT mice (*n* = 10 neurons from 4 mice) were 57.09 ± 7.16 pA and 40.16 ± 3.10 pA, respectively. The amplitudes of AMPA and NMDA EPSCs in the KO mice (*n* = 11 neurons from 6 mice) were 73.45 ± 6.25 pA and 37.56 ± 4.22 pA, respectively. Right, The AMPA/NMDA ratio in the D2-MSNs of the KO mice (2.32 ± 0.38; *n* = 11 neurons from 6 mice) did not differ significantly from that of the WT mice (1.46 ± 0.18; *n* = 10 neurons from 4 mice; *U* = 27; *p* = 0.053; Mann–Whitney *U* test). Data are presented as means ± SEMs. **p < 0.01.

Next, the holding potential was changed to +40 mV, and the EPSCs, which contained both AMPA and NMDA receptor-mediated components, were measured. Subsequently, AMPA receptor-mediated components were isolated by application of an NMDA receptor antagonist (d-AP5, 25 µM), and NMDA receptor-mediated components were obtained by electrical subtraction of AMPA components from the mixed EPSCs (Fig. 5*A*,*B*). To obtain insights into the synaptic functions of MSNs, the AMPA/NMDA ratio was calculated. In the D1-MSNs, the AMPA/NMDA ratio was significantly higher in the KO mice (1.75 ± 0.21; *n* = 11 neurons from five mice) than in the WT mice (Fig. 5*A*; 0.85 ± 0.10, *n* = 10 neurons from three mice; *U* = 10; *p* = 0.0017; Mann–Whitney *U* test). In the D2-MSNs, the AMPA/NMDA ratio appeared to be slightly higher in the KO mice (2.32 ± 0.38; *n* = 11 neurons from six mice) than in the WT mice (1.46 ± 0.18, *n* = 10 neurons from four mice), but the decrease was not significant (Fig. 5*B*; *U* = 27; *p* = 0.053; Mann–Whitney *U* test). These results suggest that *Sulf1* disruption altered the synaptic strength in the D1-MSNs.

## Discussion

In the present study, we have clarified the physiological roles of *Sulf1* in the NAc shell by examining the electrophysiological properties of MSNs in *Sulf1* KO mice. *Sulf1* disruption caused changes in neuronal excitability and glutamatergic transmission in the MSNs of the adult mice. These data clearly demonstrate the functions of *Sulf1* in central neuronal activities and synaptic transmission in the adult brain.

We first performed multiplex in situ hybridization and showed the overlapping expression of *Sulf1*, *Drd1*, and *Drd2* in the NAc shell. As shown in Figure 1, most of the *Drd1*- or *Drd2*-expressing cells expressed *Sulf1* mRNA. Among the *Sulf1*-expressing cells, ∼60 and 40% expressed *Drd1* and *Drd2*, respectively, which is similar to the percentages reported previously (Bertran-Gonzalez et al., 2008; Thibault et al., 2013; Kupchik et al., 2015; Gagnon et al., 2017). The percentage of *Drd1/Drd2* double-positive cells in the NAc shell was ∼4% in the present study, which is lower than the percentages in some studies (17% in Bertran-Gonzalez et al., 2008; 15% in Gagnon et al., 2017) but similar to those in other studies (1.5% in Thibault et al., 2013; 1.8% in Kupchik et al., 2015; 2.6% in Miyasaka et al., 2025). The discrepancy in the coexpression percentages is likely derived from the differences in the methods and mice used in the studies as well as from the difference in the analyzed subregion of the NAc shell (Gagnon et al., 2017).

D1-MSNs and D2-MSNs in the dorsal striatum and NAc differ in their electrophysiological properties (Ma et al., 2012; Francis et al., 2015; Schier et al., 2017; Al-Muhtasib et al., 2018; Cao et al., 2018; Willett et al., 2019) in addition to their gene expression, projection, and functions. In this study, we compared the intrinsic membrane properties and excitability between the D1-MSNs and D2-MSNs of adult WT mice because we have successfully established a method for preparing brain slices containing healthy neurons from adult mice by modifying the composition of a cutting Krebs’ solution (Suzuki and Momiyama, 2021). The D1-MSNs showed more hyperpolarized resting membrane potential than that of the D2-MSNs. In addition, the D1-MSNs generated more action potentials than those of the D2-MSNs at low current injections (100–240 pA), but the number of action potentials did not increase as a consequence of the injected currents being increased. In contrast, in the D2-MSNs, the number of action potentials increased as the stimulation currents increased. The D2-MSNs showed a higher rheobase but stronger activity at large depolarizing current injections than those of the D1-MSNs. The relationship between the injection currents and action potential numbers in our present study differs from those in most of the previous studies reporting that D2-MSNs in the dorsal striatum and NAc generate action potentials at a lower threshold and are more excitable than D1-MSNs [reviewed in Gertler et al. (2008) and Kreitzer (2009)]. We wondered whether this might be due to the differences in the age of the analyzed mice because juvenile mice aged 2–4 weeks were used in most slice patch-clamp recording studies, whereas we used adult mice aged 17–27 weeks. We thus performed the analysis of juvenile mice at P28–34 and found no significant differences in membrane properties and excitability between D1-MSNs and D2-MSNs. These data imply that excitability of D1-MSNs and D2-MSNs in the NAc shell is developmentally regulated and that the differences in excitability between D1-MSNs and D2-MSNs exist in the dorsal striatum and NAc core, but not in the NAc shell. The distinct electrophysiological properties of MSNs between the dorsal striatum/NAc core and NAc shell are a novel and interesting finding that should be examined in detail in future studies.

Disruption of the *Sulf1* gene resulted in changes in neuronal excitability in the adult brain, which was more pronounced in D2-MSNs. In D2-MSNs, the rheobase was significantly smaller, and more action potentials were generated at low currents in the *Sulf1* KO mice than in the WT mice, However, at larger current injections, the number of action potentials did not increase in proportion to the magnitude of the stimulation currents in the D2-MSNs of *Sulf1* KO mice. In addition, in the D2-MSNs, attenuation of the action potentials during current injections was more pronounced in the KO mice than in the WT mice. Meanwhile, in the D1-MSNs, attenuation of the action potentials was also observed in the recordings of the *Sulf1* KO mice, though the reduction ratio was not significantly higher than in the WT mice. These data indicate that *Sulf1* disruption affects firing properties in both D1-MSNs and D2-MSNs. Reduction of the AHP amplitude at 440 pA current injection as compared with that at the rheobase in the D2-MSNs of the *Sulf1* KO mice may contribute to the slow recovery from inactivation of voltage-dependent Na^+^ channels and the consequent low-frequency firing (Cloues and Sather, 2003; Duménieu et al., 2015). The AHP is generally mediated by multiple types of potassium channels. Previous studies have reported that small-conductance calcium–activated potassium channels (SK channels) regulate the AHP and excitability of MSNs in the NAc shell (Zhang et al., 2023). Furthermore, the strong depolarization lasting during step current injections is likely due to modification of voltage-gated outward–rectifying K^+^ channels with a slower time course. Actually, phosphorylation of voltage-gated potassium channels (KCNQ channels) inhibits KCNQ-mediated currents and increases excitability in NAc MSNs (Faruk et al., 2022; Tsuboi et al., 2022). In cardiomyocytes, voltage-gated potassium currents are modulated by one of the HSPGs, glypican 1 (Souza et al., 2022). Therefore, *Sulf1*-mediated changes in signal transduction pathways may affect the activities of potassium channels, thereby modulating the neuronal excitability of MSNs in the NAc shell. It is also possible that *Sulf1* regulates MSN activity by affecting the activities of other ion channels as well. Future studies on ionic currents in the NAc MSNs of *Sulf1* KO mice will be required to understand the mechanisms underlying the changes in excitability.

Interestingly, our data on the injected current-firing rate relationship indicate that *Sulf1* disruption eliminates the differences in excitability between D1-MSNs and D2-MSNs (compare Fig. 2*C* and Fig. 4*B*). Given that *Sulf1* KO does not alter the percentages of *Drd1*^+^/*Drd*2^+^ cells in the NAc (K.M. and M.M., unpublished data), it is likely that *Sulf1* disruption changes the cellular properties that play critical roles in the regulation of neuronal excitability. Several recent studies in mice have reported that D1-MSNs and D2-MSNs in the NAc shell have distinct physiological roles in the responses to appetitive or aversive stimuli (Domingues et al., 2025), hedonic eating behaviors (Guillaumin et al., 2023), and itch signal processing (Liang et al., 2022). Therefore, *Sulf1* gene disruption may lead to impairment of NAc-mediated behaviors.

The AMPA/NMDA ratio, a metric for synaptic strength and maturation, is generally used to examine changes in excitatory synaptic transmission (Kauer and Malenka, 2007). In the NAc, it is correlated with experience-dependent synaptic plasticity and changed by means of various signals including glutamate, dopamine, serotonin, opioids, and endocannabinoid (Turner et al., 2018). To explore any changes in the excitatory synapses of the NAc of *Sulf1* KO mice, we examined glutamatergic EPSCs and calculated the AMPA/NMDA ratio. In *Sulf1* KO mice, the AMPA/NMDA ratio was significantly higher in the D1-MSNs than that in the WT mice, suggesting that *Sulf1* affects glutamatergic transmission into D1-MSNs. The AMPA/NMDA ratio was slightly higher in the D2-MSNs of the *Sulf1* KO mice than in those of the WT mice, but the difference was not statistically significant. The increase in the AMPA/NMDA ratio may be related to the changes in cell-surface expression and subunit composition of AMPA receptors in MSNs (Turner et al., 2018). Because MSNs in the NAc receive glutamatergic innervation from the ventral hippocampus, amygdala, prefrontal cortex, and thalamus (Britt et al., 2012) and because they are distinctly modulated by dopamine and serotonin (Christoffel et al., 2021), pathway-specific analysis of synaptic transmission by means of optogenetic techniques will be necessary in future studies to reveal the roles of *Sulf1* in the NAc circuit. Given that the changes in the AMPA/NMDA ratio are associated with synaptic plasticity in learning and behavioral adaptations (Turner et al., 2018), NAc-dependent behaviors to reward and aversion may be affected in *Sulf1* KO mice.

HS plays important roles in neural development, axon guidance, synaptogenesis, and neuronal functions (Holt and Dickson, 2005; Condomitti and de Wit, 2018; Zhang et al., 2018; Kamimura and Maeda, 2021). In conditional *Ext1* KO mice, in which HS is not synthesized in the excitatory neurons in the forebrain, autism-like deficits were observed as a result of attenuation of EPSCs in the amygdala, presumably owing to the reduction in the number of postsynaptic AMPA receptors (Irie et al., 2012). Acute enzymatic digestion of HS resulted in impairment of long-term potentiation in hippocampal slices and reduced excitability of CA1 pyramidal neurons (Minge et al., 2017). These data suggest that HS is essential for neurotransmission and plasticity. It was also reported that *Sulf1* KO mice show a reduced hippocampal spine density and impaired long-term potentiation evoked by theta burst stimulation of Schaffer collaterals in hippocampal slices, while *Sulf1* mRNA is not detected in the adult hippocampus, and thus developmental defects are discussed as causes of these abnormalities (Kalus et al., 2009). *Sulf1/2* double KO mice have defects in the axon guidance of the corticospinal tract, but no brain defects are found in single *Sulf1* KO mice as far as we have investigated. However, because it is impossible to exclude the possibility that undetected abnormalities are the cause of the changes in the excitability of the adult NAc MSNs, mice in which the *Sulf1* gene is conditionally disrupted in the adult brain should be used in future studies.

Although we have provided compelling evidence for the involvement of *Sulf1* in neuronal functions in the adult mammalian brain, the molecular and cellular mechanisms underlying the changes observed in *Sulf1* KO mice remain to be clarified. As for the changes in synaptic transmission, SK channels regulate the AMPA/NMDA ratio as well as surface AMPA receptor expression in MSNs (Zhang et al., 2023). In addition, in a mouse model of Sanfilippo syndrome, in which HS accumulates as a result of the defects of an HS-degrading enzyme, HS from the cerebral cortex of mutant mice showed enhanced ability to increase the AMPA receptor GluA2 subunit on the cell surface (Dwyer et al., 2017). Furthermore, because diffusible secreted proteins, including Wnts, fibroblast growth factors, netrins, semaphorins, and sonic hedgehog, which are also well known axon guidance molecules (Yuzaki, 2018; Rennich et al., 2023), interact with HS, their signaling may be affected in *Sulf1* KO mice. Therefore, *Sulf1* disruption may alter AMPA receptor trafficking and synapse formation, thereby affecting the AMPA/NMDA ratio. It will be intriguing to examine the possible behavioral abnormalities that can be caused as a consequence of the changes in MSN excitability and glutamatergic transmission, for example, deficits in reward and aversive learning, in *Sulf1* KO mice in future studies.

## Synthesis

Reviewing Editor: Katrina Choe, McMaster University Decisions are customarily a result of the Reviewing Editor and the peer reviewers coming together and discussing their recommendations until a consensus is reached. When revisions are invited, a fact-based synthesis statement explaining their decision and outlining what is needed to prepare a revision will be listed below. The following reviewer(s) agreed to reveal their identity: Corey Baimel. Note: If this manuscript was transferred from JNeurosci and a decision was made to accept the manuscript without peer review, a synthesis may not be available.

The authors have done a thorough job addressing the previous reviewers' concerns. Much of the original reviews focused on data analysis and statistics, and the authors have made substantial changes to both to improve the manuscript. There are, however, remaining issues that need to be addressed.

1. The membrane properties of wildtype D1- and D2-MSNs reported here seem to be different from previous reports. The authors suggest that this is due to differences in the age of the animals and reference that many previous studies have characterized these properties in juvenile (35-45 day old) mice. This may be true, but authors should provide own data to confirm this, for example by making recordings in mice of that age in their hands.

2. The authors state that the effects of Sulf1 are well known in development, but the functions in the adult brain are not understood. They claim to examine those functions here, but I am not sure that is the best description of the work. The authors are not using a conditional model to knock out Sulf1 in adulthood, but rather knocking it out from the embryonic stage, and because of this it seems impossible to determine if the effects observed are due to the function of Sulf1 in adulthood, or rather the long-term consequences of early knock out of Sulf1. This should be addressed as a limitation of the study.

## Author Response

We would like to thank the editor and reviewers for their re-evaluation of our manuscript and for their recommendation of resubmission of the revised manuscript. We have performed additional analyses of juvenile mice, created a figure and table, and revised the manuscript to address the reviewers' concerns. We think that these revisions have improved the previous manuscript greatly and answered the reviewers' questions and concerns. We thus resubmit the revised manuscript to eNeuro for publication. The modified parts are highlighted in blue in the revised manuscript.

Point-to-point responses are listed below.

Point 1 The membrane properties of wildtype D1- and D2-MSNs reported here seem to be different from previous reports. The authors suggest that this is due to differences in the age of the animals and reference that many previous studies have characterized these properties in juvenile (35-45 day old) mice. This may be true, but authors should provide own data to confirm this, for example by making recordings in mice of that age in their hands.

Answer: Thank you for this important point. Following the comments, we have performed electrophysiological recording of wild-type juvenile mice. We injected AAV virus into the NAc of the D1-Cre and D2-Cre mice at postnatal day 20 and performed slice patch-clamp recording at postnatal days 28-34 by means of the same method used in the adult mice. As a result, we found no significant differences in the resting membrane potential, membrane resistance, and number of action potentials after current injection. These data implicate 2 important points: (1) Excitability of D1-MSNs and D2-MSNs in the NAc shell is developmentally regulated and changes as they mature. (2) Even at the juvenile age, the current-action potential relationship of D1-MSNs and D2-MSNs in the NAc shell differs from those reported in D1-MSNs and D2-MSNs in the dorsal striatum and NAc core.

We have added 2 figures (Figure 2-1 and Figure 2-2), and description in the Materials and Methods (page 9, line 143; page 12, lines 205-207; page 12, line 211 to page 13, line 212), Results (page 20, line 352 to page 21, line 365), Discussion (page 28, line 495 to page 29, line 504), and figure legends (page 53, line 942 to page 54, line 961).

Point 2 The authors state that the effects of Sulf1 are well known in development, but the functions in the adult brain are not understood. They claim to examine those functions here, but I am not sure that is the best description of the work. The authors are not using a conditional model to knock out Sulf1 in adulthood, but rather knocking it out from the embryonic stage, and because of this it seems impossible to determine if the effects observed are due to the function of Sulf1 in adulthood, or rather the long-term consequences of early knock out of Sulf1. This should be addressed as a limitation of the study.

Answer: Thank you for this important point. We have now added this in the section describing the limitations of our study (page 33, lines 577-581).
