# Supplementary figures and images for: Altered Excitability and Glutamatergic Synaptic Transmission in the Medium Spiny Neurons of the Nucleus Accumbens in Mice Deficient in the Heparan Sulfate Endosulfatase Sulf1
Source: eNeuro. 2026 Jan 2;13(1):ENEURO.0088-25.2025. doi: 10.1523/ENEURO.0088-25.2025 (PMC12768530; doi:10.1523/ENEURO.0088-25.2025)

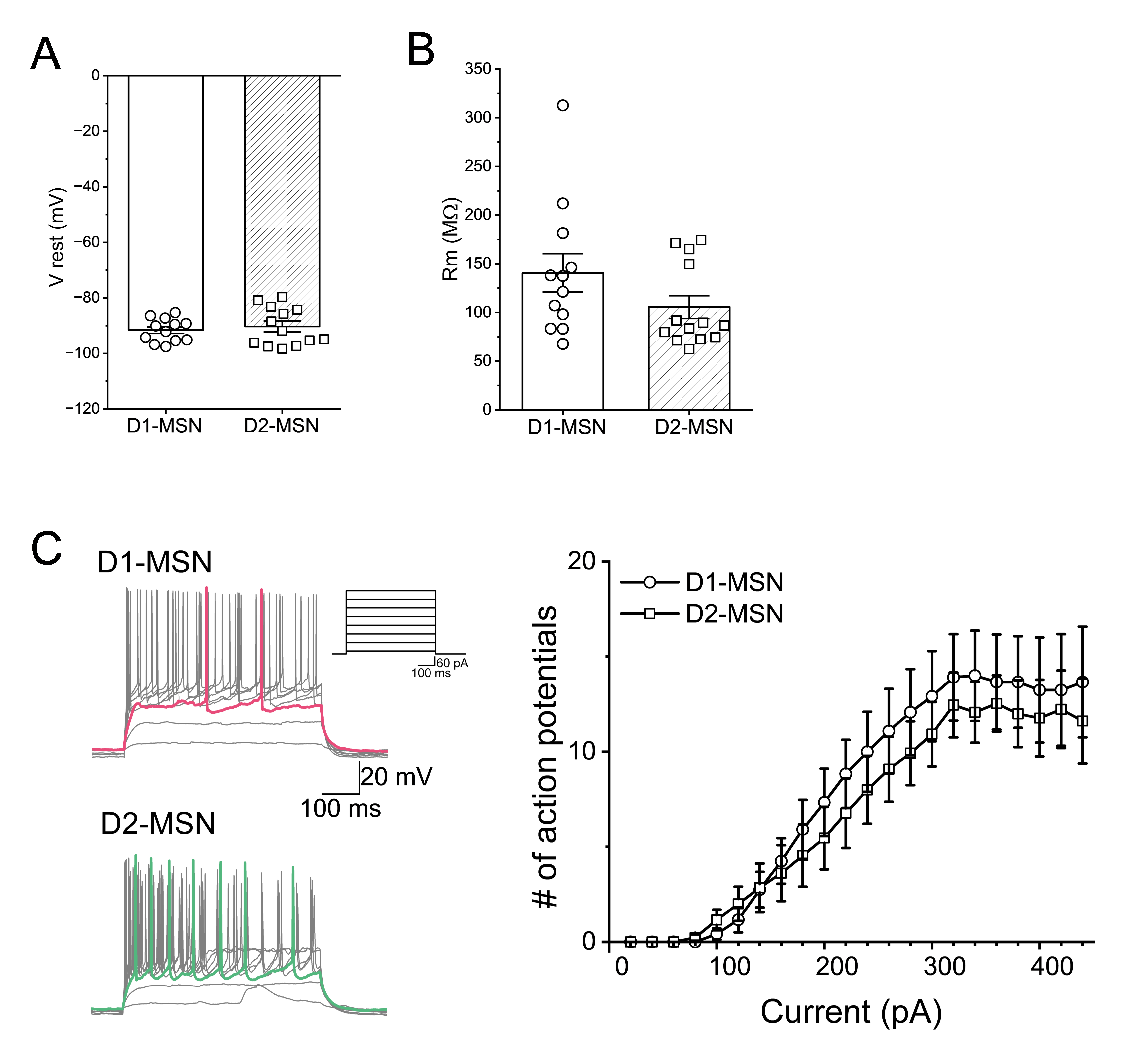

Supplement: Figure 2-1 — Comparison of electrophysiological properties between D1-MSNs and D2-MSNs of juvenile WT mice. A, Resting membrane potential (V rest). The V rest did not differ between the D1-MSNs (−91.61 ± 1.21 mV, n = 12 neurons from 3 mice) and D2-MSNs (−90.30 ± 1.89 mV, 13 neurons from 3 mice; U = 71, p = 0.72; Mann-Whitney U test). Open circles and squares indicate D1-MSNs and D2-MSNs, respectively (same as below). B, Membrane resistance (Rm). The Rm did not differ between the D1-MSNs (140.73 ± 19.78 MΩ, n = 12 neurons from 3 mice) and D2-MSNs (105.58 ± 11.76 MΩ, n = 13 neurons from 3 mice; U = 52, p = 0.17; Mann-Whitney U test). C, Left, action potentials of D1-MSNs (top) and D2-MSNs (bottom) in juvenile WT mice. Membrane potential changes were elicited by depolarizing currents (+20 to +440 pA in 20 pA increments, 500-ms duration). Scale bars, 100 ms and 20 mV. The traces when action potentials were generated at a minimum injection current are shown in color (magenta in D1-MSNs and green in D2-MSNs). The upper right panel shows a part of the current injection protocol from +20 pA to +440 pA in 60-pA increments (scale bars, 100 ms and 60 pA). Right, relationship between injected currents and number of action potentials. They did not differ significantly between the groups (main effect of cell type, F(1,23) = 0.42, p = 0.53; main effect of current, F(21,483) = 33.8, p < 0.0001; interaction; F(21,483) = 0.31, p = 0.999). Download Figure 2-1, TIF file. [file eneuro-13-ENEURO.0088-25.2025-s001.tif]
