# Supplementary material for: Altered Excitability and Glutamatergic Synaptic Transmission in the Medium Spiny Neurons of the Nucleus Accumbens in Mice Deficient in the Heparan Sulfate Endosulfatase Sulf1
Source: eNeuro. 2026 Jan 2;13(1):ENEURO.0088-25.2025. doi: 10.1523/ENEURO.0088-25.2025 (PMC12768530; doi:10.1523/ENEURO.0088-25.2025)
Supplement: Figure 2-2 — Results of statistical analyses of juvenile mouse studies in Figure 2-1. Download Figure 2-2, DOCX file. [file eneuro-13-ENEURO.0088-25.2025-s002.docx]

| Figure 2-2 Results of statistical analyses of juvenile mouse studies in Figure 2-1 | | |  |  |
| --- | --- | --- | --- | --- |
| Figure | Data structure | Type of test | Sample size | Statistical data |
| Figure 2-1 |  |  |  |  |
| Figure 2-1A  Resting membrane potential (V rest) D1-MSN vs D2-MSN | unknown | Mann–Whitney U test | D1-MSN, n = 12 D2-MSN, n = 13 | U test, D1-MSN vs D2-MSN U = 71, p = 0.72 |
| Figure 2-1B  Membrane resistance (Rm) D1-MSN vs D2-MSN | unknown | Mann–Whitney U test | D1-MSN, n = 12 D2-MSN, n = 13 | U test, D1-MSN vs D2-MSN U = 52, p = 0.17 |
| Figure 2-1C  Number of action potentials  D1-MSN vs D2-MSN | Normally distributed | 2-way mixed model ANOVA | D1-MSN, n = 12 D2-MSN, n = 13 | cell type, F(1,23) = 0.42, p = 0.53 current, F(21,483) = 33.8, p < 0.0001 interaction, F(21,483) = 0.31, p = 0.999 |
